# A miRNA Signature of Chemoresistant Mesenchymal Phenotype Identifies Novel Molecular Targets Associated with Advanced Pancreatic Cancer

**DOI:** 10.1371/journal.pone.0106343

**Published:** 2014-09-03

**Authors:** Alakesh Bera, Kolaparthi VenkataSubbaRao, Muthu Saravanan Manoharan, Ping Hill, James W. Freeman

**Affiliations:** 1 Department of Medicine, Division of Hematology and Oncology, University of Texas Health Science Center at San Antonio, San Antonio, Texas, United States of America; 2 Cancer Therapy and Research Center, Experimental and Developmental Therapeutics Program, San Antonio, Texas, United States of America; 3 Research and Development, Audie Murphy Veterans Administration Hospital, San Antonio, Texas, United States of America; University of Kansas School of Medicine, United States of America

## Abstract

In this study a microRNA (miRNA) signature was identified in a gemcitabine resistant pancreatic ductal adenocarcinoma (PDAC) cell line model (BxPC3-GZR) and this signature was further examined in advanced PDAC tumor specimens from The Cancer Genome Atlas (TCGA) database. BxPC3-GZR showed a mesenchymal phenotype, expressed high levels of CD44 and showed a highly significant deregulation of 17 miRNAs. Based on relevance to cancer, a seven-miRNA signature (miR-100, miR-125b, miR-155, miR-21, miR-205, miR-27b and miR-455-3p) was selected for further studies. A strong correlation was observed for six of the seven miRNAs in 43 advanced tumor specimens compared to normal pancreas tissue. To assess the functional relevance we initially focused on miRNA-125b, which is over-expressed in both the BxPC3-GZR model and advanced PDAC tumor specimens. Knockdown of miRNA-125b in BxPC3-GZR and Panc-1 cells caused a partial reversal of the mesenchymal phenotype and enhanced response to gemcitabine. Moreover, RNA-seq data from each of 40 advanced PDAC tumor specimens from the TCGA data base indicate a negative correlation between expression of miRNA-125b and five of six potential target genes (*BAP1*, *BBC3*, *NEU1*, *BCL2*, *STARD13*). Thus far, two of these target genes, *BBC3* and *NEU1*, that are tumor suppressor genes but not yet studied in PDAC, appear to be functional targets of miR-125b since knockdown of miR125b caused their up regulation. These miRNAs and their molecular targets may serve as targets to enhance sensitivity to chemotherapy and reduce metastatic spread.

## Introduction

PDAC continues to have the worst prognosis of any solid tumor. In 2013, it is estimated that more than 45,000 new cases will be diagnosed in the United States with mortality to incidence ratio almost equal to one [1]. Gemcitabine is the most commonly used chemotherapy for pancreatic cancer but is significantly metabolized in plasma and therefore requires high doses leading to toxicity [2]. Many PDAC are initially resistant to gemcitabine and responsive ones generally develop resistance during the course of treatment [3].

It remains to be determined whether the chemoresistant phenotype is induced as a result of therapy or whether there is a subpopulation of cancer cells within the tumor that are innately more resistant to therapy. Recent evidence indicates that most solid tumors, including PDAC possess a distinct subpopulation of cancer stem cells (CSCs). Evidence also suggests that CSCs are inherently more resistant to chemotherapy and utilize their self-renewal potential to regenerate new tumor growth [4]. A previous study links CSCs with epithelial to mesenchymal transition (EMT) [5].

EMT represents a trans-differentiation program that is required for tissue morphogenesis during different cellular developmental progression [6]. The EMT process can be regulated by a diverse array of cytokines and growth factors, such as TGF-β, whose activities are deregulated during tumor progression [7]. EMT induction in cancer cells results in the acquisition of invasive and metastatic properties. Studies also indicate that EMT also contributes to chemoresistance and that these cells possess CSC markers [8]. The development of chemoresistance in cancer cells to anticancer drugs including gemcitabine may be regulated or contributed to by micro-RNAs (miRNAs). miRNAs are small non-coding RNA molecules (22 nucleotides), which play crucial roles in transcriptional regulation of gene expression. The development of chemoresistance through an increase in the number of CSCs has been attributed to alterations at the level of miRNAs in pancreatic and other solid tumors [9].

Recent findings indicate an interrelationship between miRNAs, EMT phenotype, CSCs and chemoresistance [10], [11]. Studies also suggest that miRNAs play a role in regulating the EMT process [3], [11]. For instance, miRNAs have been shown to drive EMT by regulating cadherin 1 and additional EMT related molecules [12]. MiR-200a, miR-200b and miR-200c were down regulated in gemcitabine-resistant pancreatic cancer cells [13]. Studies also indicate that re-expression of the miR-200 family of miRNAs up regulated cadherin 1 and down regulated ZEB1 and vimentin [14]. Additionally, cancer cells with an EMT phenotype and that were resistant to gemcitabine showed down regulation of let-7 members [5], [15], [16]. In vitro studies of PDAC indicate a linkage among EMT, invasiveness and resistance to chemotherapy and other studies support the notion that miRNAs play a role in these processes by regulating gene expression [3], [11].

In this study we sought to identify a miRNA signature for PDAC cells that possess chemoresistant and a mesenchymal phenotype (CRMP). Because patients with advanced PDAC tend to show minimal response to chemotherapy we further determined whether this miRNA signature might be reflected in their tumor tissues. These studies show that gemcitabine treatment induces a population of cells with CRMP in vitro and that a miRNA signature selected for this CRMP is shared with tumors specimens from advanced PDAC. Moreover, this study was able to initially establish a functional relevance of one over expressed miRNA (miR-125b) from this signature and potential tumor suppressor target genes. This study provides the basis for further dissecting the roles of miRNAs in CRMP. These miRNAs and their molecular targets may provide novel therapeutic strategies to eliminate a tumor cell population that is generally resistant to gemcitabine and possibly other chemotherapy.

## Materials and Methods

### Materials

Gemcitabine purchased from LKT Laboratories, Inc (St. Paul, MN) was dissolved in sterile PBS solution. Reverse transcription reagents and TaqMan real-time PCR master-mix were purchased from Applied Biosystems/Life Technologies (Foster city, CA). All other chemicals were obtained from Sigma–Aldrich (St. Louis, MO).

### Cell lines

The human pancreatic adenocarcinoma cell line BxPC3, Capan-2, AsPC-1 and Panc-1 were purchased from ATCC and cultured them in DMEM media (except BxPC3) with 1% penicillin/streptomycin and 10% fetal bovine serum (FBS) in a humidified incubator containing 5% CO_2_ at 37°C. BxPC3 was cultured in RPMI media with 1% penicillin/streptomycin and 10% FBS. This PDAC cell line BxPC3 was transiently exposed for four hours every seven days with increasing concentrations (50 ng to 1.5 µg/ml) of gemcitabine over a six-week period. The resulting gemcitabine resistant cell line referred to as BxPC3-GZR. For maintenance, BxPC3-GZR cells were expanded in culture medium and frozen in aliquots. For experiments BxPC3-GZR cells were thawed and allowed to expand in culture for two days and then treated with gemcitabine (1.5 µg/ml) for four hours. Gemcitabine was removed and BxPC3-GZR cells were harvested the following day for experiments including quality control Westerns blots to show that the acquired EMT phenotype was maintained.

### Western blots analyses and immunofluorescence staining

Western blot analysis was performed as described previously [17]. Primary antibodies used were as follows: E-cadherin and Neu1 from Santa Cruz Biotechnology, Inc. (Santa Cruz, CA); CD44, beta-actin and PUMA (*BBC3*) were purchased from Cell Signaling Technology (Beverly, MA), vimentin from Life Technologies (Carlsbad, CA). Horseradish peroxidase-conjugated secondary antibodies were purchased from Amersham Biosciences (Piscataway, NJ). For immunofluorescence (IF) staining, cells grown on round cover slips in a 24-well plate. Fixing and immunostaining followed by capturing images were performed as described previously [18].

### Matrigel Cell Invasion Assays

The invasive behavior of cells was analyzed by Matrigel invasion assays as described previously [17], [18].

### The expression profiling of miRNAs

Total RNA including small non-coding miRNA was isolated from BxPC3 and BxPC3-GZR using mirNeasy kit (Qiagen) per manufacturer procedures. Expression analysis of miRNA in both control BxPC3 and BxPC3-GZR cells were performed by LC Sciences (Houston, TX) using a proprietary μParaflo microfluidic biochip technology. Significant changes in miRNA expression were analyzed by t-test as provided by LC Sciences (Houston, TX). Only the most highly significant changes of both over and under expressed miRNAs were considered for further analysis (Table 1).

**Table 1:** The list of miRNAs that are highly significant in terms of expression in the gemcitabine resistant BxPC3-GZR cells compared to control BxPC3 cells.

miRNA ID	p Value	Log2 (R/Wt)
***Positively correlated miRNAs (Bx-GZR> BxPC3)***		
hsa-miR-125b	0.004	1.78
hsa-miR-155	0.00427	1.48
hsa-miR-100	0.00482	1.55
†hsa-miR-4324	0.00676	2.93
hsa-miR-21	0.00237	0.8
hsa-miR-15b	0.00388	1.19
hsa-miR-25	0.00516	0.53
†hsa-miR-424*	0.00756	0.57
has-miR-99b	0.00379	1.19
***Negatively correlated miRNAs (Bx-GZR< BxPC3)***		
hsa-miR-1246	0.00007	−4.03
hsa-miR-205	0.00445	−2.14
hsa-miR-4443	0.00504	−1.27
hsa-miR-30d	0.00185	−0.83
hsa-miR-27b	0.00877	−0.61
hsa-miR-4485	0.00456	−0.81
†hsa-miR-378*	0.00649	−0.96
hsa-miR-455-3p*	0.00485	−0.9

† These transcripts are statistically significant but very low signal.

### Reverse transcription and TaqMan quantitative PCR

TaqMan gene-expression assays (Life Technologies, Carlsbad, CA) were used to quantify the expression levels of both mRNA and mature miRNA. Total RNA extracted by mirVana (Life Technologies, Carlsbad, CA) RNA isolation kit and followed by reverse transcribed in a reaction mixture containing random hexamer primers (for mRNA RT-PCR) and miR-specific stem-loop RT primers (for miRNA RT-PCR). Quantitative PCR was performed in triplicate with reactions containing amplified cDNA and TaqMan primers in Universal Master Mix without AmpErase UNG (Applied Biosystems, Carlsbad, CA) by following manufacturer's protocol. All mRNA data and miRNA data are expressed relative to 18S and U6 respectively by TaqMan PCR performed on the same samples, unless otherwise specified. Fold expression was calculated from the triplicate of C_T_ values following the 2^−ΔΔC^_T_ method.

### Stable cell line generation for miR-125b knockdown

Gemcitabine resistant BxPC3-GZR and Panc-1 cells were used in the miR-125b knock down study. System Bioscience's (SBI, Mountain View, CA) miRZipTM anti-sense miRNAs are stably expressed RNAi hairpins that inhibit miRNA activity. The miRZip shRNAs produce short, single-stranded anti-miRNAs that competitively bind their endogenous miRNA target and inhibit its function. The miRZip short hairpin RNAs are cloned into SBI's pGreenPuroTM shRNA expression vector, an improved third generation HIV-based expression lenti-vector. The lentiviral vector contains the genetic elements responsible for packaging, transduction, and stable integration of the viral construct into genomic DNA, inducing expression of the anti-miRNA effector sequence. For production of a high titer of viral particles, we used the ViraPowerTM Lentiviral Support Kit (Invitrogen, Carlsbad, CA) employing LipofectamineTM 2000 (Invitrogen) for transfecting the miRzip vectors into HEK-293T cells. Because infected cells stably express GFP and puromycin, as well as the anti-miRNA cloned into the miRZipTM vector, we used puromycin to select for the infected cells harboring the miRzip. After screening we isolated BxPC3-GZRΔmiR-125b or Panc-1ΔmiR-125b cells. In the similar way we also generated Zip control of both cell-lines with miRZipTM vector. For determining drug sensitivity, cells were treated with indicated concentrations of gemcitabine for 96 h and MTT assays were performed as previously described [19].

### MiRNA and transcriptome profiling of tumor data from The Cancer Genome Atlas (TCGA)

*miRNA:* Level 3.1.1.0 miRNA sequence data from TCGA data portal was downloaded for 43 tumor samples with one normal tissue. A data matrix was prepared by combining the raw read counts of 1046 miRNAs from 44 [43 tumor and 1 normal] samples. Based on the assumption that miRNA sequence data follow a negative binomial distribution [20], [21] the read counts were size-factor normalized using DESeq version 1.10.1 [22] package in R version 2.15.3. Normalized read count data was used to compute log2 fold change between tumor and normal samples.

*Transcriptome:* Level 3.1.1.0 RNA sequence data from TCGA data portal was downloaded for 40 tumor samples with one normal tissue as control. We used RSEM software for determining quantity of transcripts from RNA-Seq data. RSEM upper quartile normalized read counts data from 41 (40 tumors and 1 normal) tissue samples were used to compute log2 fold change between tumor and normal samples.

### Statistical analysis

The Student's unpaired t-test was used to compare individual group means. A p-value of <0.05 was considered as statistically significant. All values in the figures and text were expressed as the mean ± S.D.

## Results

### Generation of a pancreatic cancer CRMP cell line model

**Figure 1:**
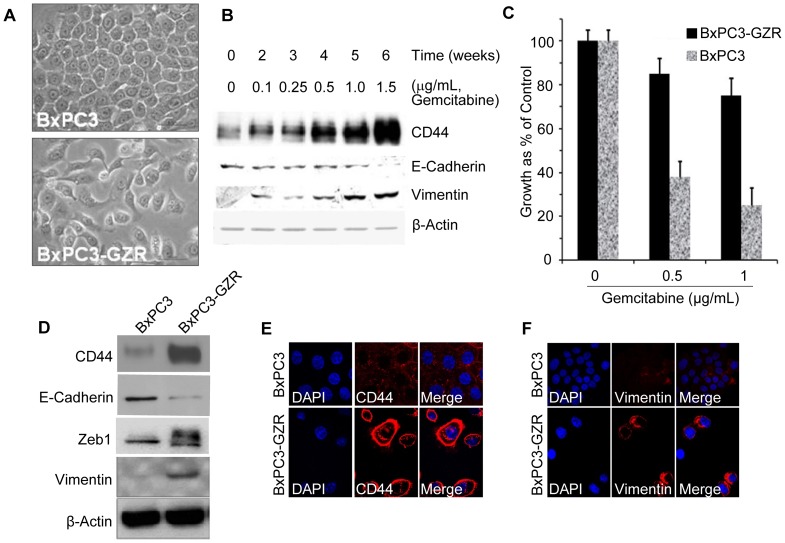
Characterization of BxPC3-GZR, a cell line model for CRMP. **A.** Morphological differences between parental BxPC3 and chemoresistance mesenchymal BxPC3-GZR cells. **B.** Western blot showing that BxPC3-GZR cells possess an EMT phenotype, which is demonstrated by increased expression of vimentin and a decrease of E-cadherin and express the stem cell marker CD44. **C.** MTT assay comparing the growth of the parental BxPC3 and BxPC3-GZR at 96 hrs after treatment with different concentrations of gemcitabine. **D**. Western blot comparing the expression of CD44, E-cadherin, Zeb-1 and vimentin after four passages of BxPC3 and BxPC3-GZR. **E** and **F**. Immunofluorescence confocal microscopy images show the differential expression of CD44 and vimentin in BxPC3 and BxPC3-GZR cells.

The PDAC cell line BxPC3 was transiently exposed to increasing concentrations of gemcitabine over a six-week period. The resulting gemcitabine resistant BxPC3-GZR cells were compared with the parental BxPC3 cells for differences in morphology, response to gemcitabine, expression of mesenchymal, epithelial markers and CD44. Comparisons for morphology show that the parental BxPC3 cells grew in tightly packed areas and showed a flat and rounded appearance, characteristic of an epithelial like morphology; whereas, BxPC3-GZR cells grew as loosely-associated cells with a spindle-like morphology characteristic of a mesenchymal phenotype (Fig. 1A). The sensitivity to treatment with gemcitabine was compared between BxPC3-GZR and parental BxPC3 cells. BxPC3-GZR cells showed greater than a twofold decrease in response to gemcitabine compared to its parental cells BxPC3 (Fig. 1C). To determine whether BxPC3-GZR cells are also cross resistant to another chemotherapeutic compound, cells were treated with paclitaxel and MTT assays were performed. While BxPC3-GZR cells showed more than a twofold decrease in sensitivity to gemcitabine, these cells showed only a modest decrease in sensitivity to paclitaxel (Fig. S1). These observations suggest that different signaling pathways may be responsible for resistance against different drugs. A recent study with side population of PDAC cells with properties of cancer stem cells and that were selected for resistance to gemcitabine were not resistant to 5-FU [23]. Western blot analysis of cells collected at each week over the six weeks of increasing gemcitabine treatment revealed that the level of epithelial marker E-cadherin gradually decreased with concomitant increase in the levels of mesenchymal marker vimentin and the stem cell marker CD44 (Fig. 1B).

More importantly, we have also monitored and compared the expression levels of same proteins of BxPC3 and BxPC3-GZR cells that where expanded and passed for successive generations in culture. The stability of the BxPC3-GZR phenotype was confirmed in short term cultures with BxPC3-GZR cells showing a lower level of E-cadherin, up regulation of vimentin expression and elevated expression of stem cell markers CD44 (Fig. 1D). In agreement with the Western blot data, immunofluorescence analysis indicated a much stronger staining of both CD44 and vimentin in BxPC3-GZR cells compared to the parental BxPC3 counterpart (Fig. 1E, F).

### Identification of a miRNA signature associated with CRMP in PDAC

**Figure 2:**
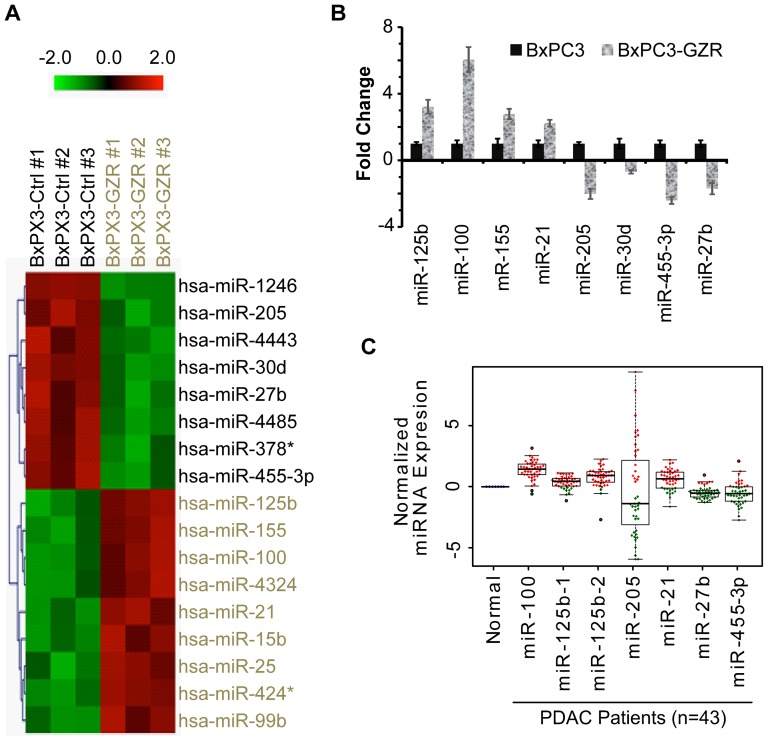
miRNA signature of BxPC3-GZR cells. **A.** Heat map data analysis of the miRNA microarray assays comparing BxPC3 parental cells and drug resistant BxPC3-GZR cells. Only miRNAs were chosen which had significantly over or under expressed as compared to parental cells (high fold changes based on log2 values and very low p-values) were taken for further validation. The statistical values are represented in the *Table 1*. **B.** Validation of miRNA microarray data was done for eight differentially expressed miRNAs using TaqMan qPCR assay. In each cell line, the expression level of indicated miRNA was compared between parental BxPC3 cells and BxPC3-GZR cells. RNU43 or U6 was used as an internal miRNA control. **C.** TCGA data analysis showing that 6 of the 7 miRNAs validated for being deregulated in BxPC3-GZR cells also showed differential expression in tumor specimens from patients with advanced PDAC. Tumor specimens from 43 patients with advanced PDAC were analyzed for miRNA expression compared to normal pancreas tissue.

Studies indicate that various miRNAs such as miR-100, miR-21, let-7, miR- 34a and miR - 200c play critical role in regulating tumorigenesis and chemoresistance in different cancers including pancreatic cancer [24]–[26]. Studies also showed a role for miRNAs in development of drug resistance in a variety of malignancies [11]. The expression level of miRNA was compared in BxPC3 and BxPC3-GZR cells by μParaflo microfluidic chip miRNA profiling. After calculation and eliminating non-significant miRNAs (in terms of expression), a highly significant miRNA profile was established that distinguished BxPC3-GZR cells from parental BxPC3 cells (Fig. 2, *Table 1*). This profile showed most significant nine miRNAs that were up regulated in BxPC3-GZR cells compared to BxPC3 (miR-125b, miR-155, miR-100, miR-4324, miR-21, miR-15b, miR-25, miR-424*, miR-99b) and eight that were down regulated (miR-1246, miR-205, miR-4443, miR-30d, miR-27b, miR-4485, miR-378*, miR-455-3p).

Based on relevance to cancer and chemoresistance, seven of these miRNAs were selected for further study. Real Time–PCR using TaqMan assays were done to validate over or under-expressed miRNAs identified by miRNA arrays (Fig. 2B). Finally we identified four of the over-expressed miRNAs (miR-125b, miR-155, miR-100, miR-21) and three under expressed miRNAs (miR-27b, mir-205, and miR-455-3p) by miRNA microarrays were validated by Real-Time PCR (Fig. 2B).

### The miRNA signature from the CRMP cell line model is also detected in clinical specimens from patients with advanced PDAC

To establish the potential relevance of the validated CRMP miRNA signature with miRNAs found in advanced PDAC, whole transcriptome analyses (miRNA and mRNA-seq, expression levels) were performed with the PDAC patient specimen data from the TCGA data sets. In the TCGA database there are a total 43 PDAC patients samples containing expression level of miRNAs with a normal pancreas tissue. Forty of these patients' tumor specimens had data for both mRNA and miRNA expression levels. A comparison of the over and under expressed miRNAs in the TCGA with those in our miRNA array is presented in *Table 2*. As shown in *table 2*, of the seventeen miRNAs comprising the miRNAs aberrantly deregulated in BxPC3-GZR (9 over expressed and 8 under expressed) miRNA data for 14 of these were available in the TCGA analyses and for all seven of the validated miRNAs. A further comparison was done on the six-miRNA signature validated from the array data and for which there were matching data for advanced PDAC specimens. An analysis of this data is shown in Figure 2C. Interestingly there was a significant correlation with common expression levels of miRNAs in advanced PDAC tumor specimens with the validated CRMP miRNA signature for five of the six miRNAs (Fig. 2C, *Table 2*). It is important to note that the miR-125b data for the tumor specimens is given as both miR-125b-1 and miR-125b-2 isoforms, whereas in cell lines we only determined miR-125b-1 isoform (designated as miR-125b). Interestingly, the precursors of miR-125b-1 and miR-125b-2 isoforms were known to originate independently from different chromosomal loci although their mature sequences and targets are same [27]. The most notable miRNAs over expressed in both BxPC3-GZR and clinical specimens were miR-100, miR-21, miR-125b-1, miR-125b-2 whereas miR-205, miR-27b and miR455 were under-expressed.

**Table 2:** Comparison and validation of in vitro data with the patient samples.

Name of miRNA	Expression in patient samples relative to normal	
	*(n = 43 TCGA Samples)*	
	# of *Over* expressed	# of *Under* expressed
**Over expressed in BxPC3-GZR**		
hsa-miR-125b-1	34	9
hsa-miR-125b-2	39	4
hsa-miR-155	16	27
hsa-miR-100	41	2
hsa-miR-4324	ND*	ND
hsa-miR-21	31	12
hsa-miR-25	32	11
hsa-miR-99b	27	16
**Under expressed in BxPC3-GZR**		
hsa-miR-1246	ND	ND
hsa-miR-205	18	25
hsa-miR-4443	NA	NA
hsa-miR-30d	41	2
hsa-miR-27b	7	36
hsa-miR-4485	NA	NA
hsa-miR-378	10	33
hsa-miR-455	11	32

TCGA data was analyzed to determine the differential expression of different miRNAs indentified in BxPC3-GZR cells.

*ND = Not detected (very low copy number). NA = these micro-RNA data are not available in the database.

### miR-125b plays a role in regulating the CRMP in PDAC

**Figure 3:**
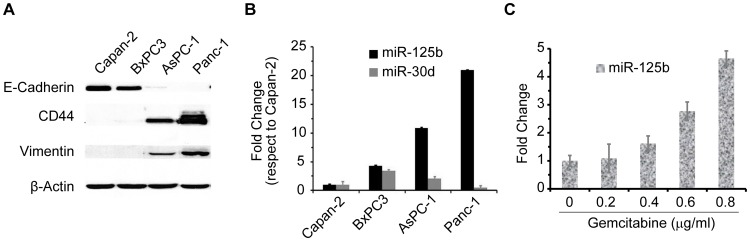
Micro-RNA-125b partially regulates CRMP. **A.** Western blot analyses showing the expression levels of EMT markers in PDAC cells in which miR-125b expression was determined. **B.** TaqMan qPCR analysis showing the relative expression of miRNAs (miR-125b and miR-30d) in different PDAC cells. MiR-30d is used as a control. **C.** Induction of miR-125b expression in BxPC3 upon treatment of gemcitabine. Quantitative PCR (TaqMan) were performed after 72 hrs of incubation with the drug in order to monitor the expression level of miR-125b in BxPC3 cells. Data indicates that expression of miR-125b is increased by the treatment of gemcitabine in a dose dependent manner.

Initial studies were undertaken to determine whether the miRNAs identified in BxPC3-GZR cells and that were common to clinical specimens played a role in the CRMP. The miR-125b that was commonly over expressed in both advanced PDAC clinical specimens and in BxPC3-GZR cells (Fig. 2) was chosen for these analyses. For further studies, three PDAC cell lines (AsPC-1, Capan-2, Panc-1) in addition to BxPC3 were screened for expression levels of EMT markers along with miR-125b and miR-30d (Fig. 3 A, B). MiR-30d was used for comparison of differential expression of miRNA since it showed equal expression level in both parental (BxPC3) and resistant cells by qPCR analysis. In these cell lines the epithelial marker E-cadherin is inversely related to the expression of miR-125b; whereas, the mesenchymal marker vimentin and the stem cell marker CD44 were proportionally related to the expression of miR-125b (Fig. 3A, B). These data further suggests that the EMT phenotype may be regulated in part by the constitutive expression of miR-125b in Capan-2 and BxPC3 cells show an epithelial like phenotype and express low levels of miR-125b; however, AsPC-1 and Panc-1 cells are mesenchymal like and show higher levels of miR-125b (Fig. 3 A, B). Besides we have also monitored the expression of miR-125b in BxPC3 cells upon treatment with gemcitabine. Data indicates that 72 hours treatment with gemcitabine induced the expression of miR-125b in BxPC3 cells in a dose dependent manner (Fig. 3C). Therefore, these findings indicate that both chemoresistance and mesenchymal phenotypes are regulated by the differential expression of miR-125b.

**Figure 4:**
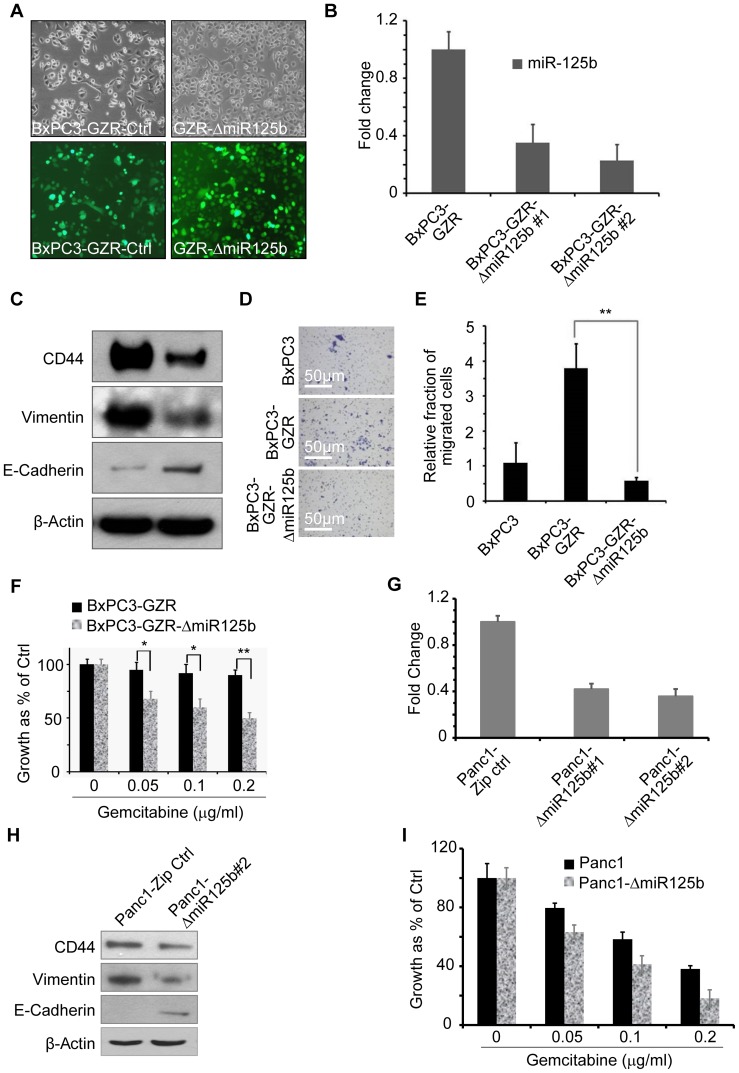
Knockdown of micro-RNA-125 reverses the mesenchymal phenotype and increases the drug sensitivity in BxPC3-GZR cells. **A.** Establish the Lenti-viral based stable cells expressing anti-miR-125b in BxPC3-GZR (Zip-control and miR-125b knock-down). **B.** TaqMan qPCR assays showing the knockdown of miR-125b in BxPC3-GZR-Zip ctrl cells compared to BxPC3-GZRΔmiR-125b cells. **C.** Expression of anti-miR-125b (Zip technology, SBI) decreases the expression of EMT and stemness marker monitored by Western bolt assays. Panels **D** and **E** show the attenuation of cell migration by knocking down miR-125b expression. Images were taken after crystal violet staining of migrated cells and the data was plotted as a graph (Bar = 50 µm). **F.** Knockdown of miR-125b increases response to gemcitabine. BxPC3-GZR-ZiP-Ctrl and BxPC3-GZRΔmiR-125b cells were treated with gemcitabine at different concentrations and the MTT assays were performed after 96 hours of treatment. **G.** Knockdown of miR-125b in Panc-1 cells. Lenti-viral based stable cells were generated to inhibit miR-125b expression (Zip technology,). TaqMan qPCR assays were performed to measure the expression of miR-125b in Zip-control Panc-1 cells and knock down cells (Panc-1 ΔmiR-125b). **H.** Inhibiting miR-125b decreases CD44 and vimentin expression while up regulating the expression of E-cadherin. **I.** Knockdown of miR-125b increases the response of Panc-1 to gemcitabine. MTT assays were done after 96 hours of gemcitabine treatment. Statistical significance values *p<0.05 and **p<0.01 were calculated using student's T-tests.

In order to determine whether miR-125b expression is directly related to CRMP, we knocked down the expression of miR-125b by using Zip technology and stable cell lines generated and assayed for expression of miR-125b in both BxPC-GZR-Zip-Ctrl and in BxPC3-GZR-ΔmiR-125b cells (Fig. 4A). The morphology of miR-125b knockdown cells was also assessed and knockdown of miR-125b restored the epithelial like morphology (Fig. 4A). TaqMan qPCR assay indicated an approximate 80% knockdown of miR-125b in BxPC3-GZR cells (Fig. 4B). Knockdown of anti-miR125b reversed EMT markers as shown by the increased expression of the epithelial marker E-cadherin and the decreased expression of the mesenchymal marker vimentin and also decreased CD44 expression (Fig. 4C). Knockdown of miR-125b decreased cell migration (Fig. 4D, E) and partially reversed the gemcitabine resistance of BxPC3-GZR cells (Fig. 4F).

In order to confirm that this effect of miR-125b knockdown was not unique to BxPC3-GZR cells, miR-125b was also knocked down in Panc-1 cells. Similar to that seen in miRNA-125b knockdowns for BxPC3-GZR cells, Western blot and MTT assay data indicated that knockdown of miR-125b expression in Panc-1 cell partially reversed the mesenchymal phenotype (Fig. 4G,H) and enhanced response to gemcitabine (Fig. 4I).

### Gene targets of miR-125b

**Figure 5:**
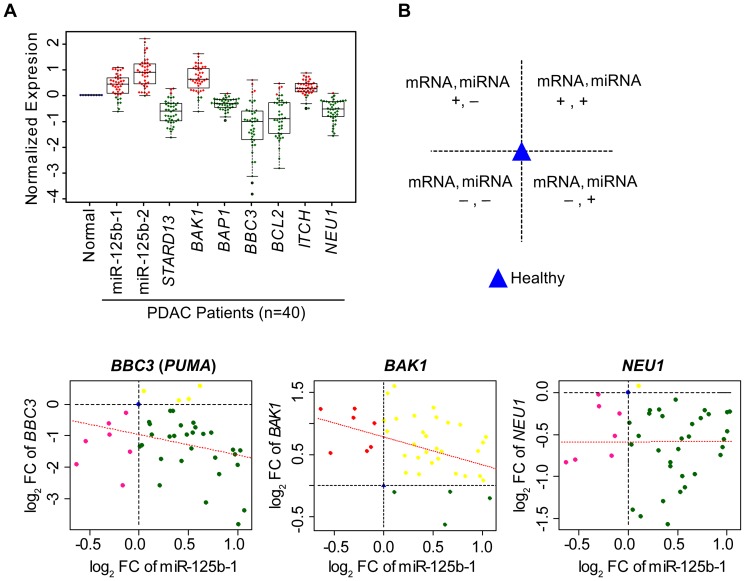
Gene targets of miR-125b. TCGA data analyses were performed with the clinical pancreatic tumor specimens (n = 40) for miRNA expression with the corresponding target mRNAs. **A.** Target mRNA expression profile analyses for miR-125b. Determined the expression of known miR-125b targets (*BBC3* (PUMA), *BCL2*, *STARD13*, *BAK1*, *BAP1*, *ITCH* and *NEU1*). **B.** A direct co-relation of the target mRNA and miR-125b expression level were measured in the same tumor from pancreatic adenocarcinoma [PDAC] patient's sample. First panel explains the different quadrants which includes up-regulation (+ Ve sign) or down regulation (**-** Ve sign) of mRNA and miRNA expression levels. Other panels are representing expression level of different mRNA (*BBC3, Neu1 and BAK1*) compared with the expression of miR-125b in the same tumor.

**Figure 6:**
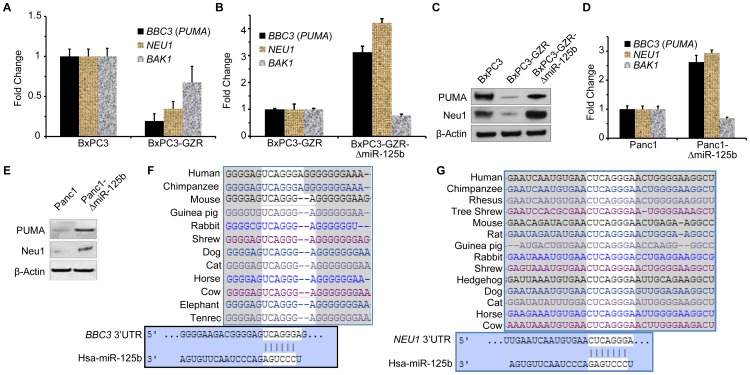
Validation of miR-125b target genes by qPCR and Western blot assays. **A.** PUMA (*BBC3*) and Neu1 are most prominent mRNA targets of miR-125b. The TaqMan qPCR analyses were performed to validate clinical data. The qPCR results indicating the expression level of different mRNA (*BBC3, Neu1 and BAK1*) which are direct target of miR-125b compared between BxPC3 parental cells and resistant GZR cells. **B.** Comparison of mRNA expression level of *BBC3, Neu1 and BAK1* in BxPC3-GZR cells and stable BxPC3-GZR cells expressing anti-miR-125b. **C.** Western blots analysis to monitor the expression level of PUMA and Neu1 in BxPC3, BxPC3-GZR, and miR-125b knockdown cells. **D** and **E**. Inhibiting miR-125b restores BBC3 and Neu-1 expression in Panc-1 cells, qPCR (D) and Western blot (E). **F, G**. Species conservation and matching of the seed sequence in 3′UTR of *BBC3* and *NEU1* mRNAs with miR-125b sequence were monitored by using Targetscan free web-based software.

Since miRNAs act by either repressing or cleaving their target mRNAs, we carried out gene expression analysis of several known downstream targets of miR-125b. Several studies indicated that mRNA targets of miR-125b are *BBC3* (PUMA), *ITCH*, *BAK1*, *BCL2*, *NEU1*, *PPP1CA*, *PPP2CA* [28] [29]; *STARD13* (DLC2) [30]; *AP1M1*, *STK11IP*, *PSMD8*, *TBC1D1*, *TDG*, *MKNK2*, *DGAT1*, BAP1, *GAB2*, *SGPL1* [28]. We analyzed the clinical tumor data for these above mRNA expression levels (Fig. 5). RNA-seq from TCGA showed that the expressions of some of these genes (*BAP1*, *BBC3* or PUMA, *BCL2*, *NEU1*, *STARD13*) are negatively correlated with the expression of miR-125b. We analyzed both the general expression trend of the targeted genes in the PDAC specimens from the TCGA database (Fig. 5A), as well as the expression level of targeted gene in relation to the expression of miRNA-125b within the same tumor specimens (Fig. 5B). Next, we have monitored the expression level of p53 up-regulated modulator of apoptosis (PUMA). PUMA also known as Bcl-2-binding component 3 (BBC3) is a pro-apoptotic protein and member of the Bcl-2 protein family [31], [32]. PUMA is involved in both p53-dependent and -independent apoptosis pathway induced by a variety of signals [33]. Recent studies also indicate that *NEU1* product Neu1 salidase is important in regulation of integrin β4-mediated signaling, leading to suppression of cancer metastasis [34]. However, a separate study indicates that Neu1 salidase enhances EGFR signaling and thus could be tumor promoting [35]. The present findings along with the web-based miRNA target scan data suggest that miR-125b directly targets the 3′UTRs of PUMA (BBC3) and Neu1 (Fig. 6. F,G). In support of this, the mRNA expressions for *BBC3* (PUMA) and *NEU1* were inversely correlated with the expression of miR-125b (Fig. 6A) in BxPC3-GzR cells.

Next, we compared the expression of three different potential miR-125b target genes PUMA (*BBC3*) (most under-expressed gene in patient samples), *NEU1* and *BAK1* (up-regulated gene) mRNAs between BxPC3 parental and BxPC3-GZR cells (Fig. 6B). In a similar manner, we also compared the expression of these genes between BxPC3-GZR cells and the BxPC3-GZR cells expressing anti-miR-125b (Fig. 6B). The data indicated that the expression of PUMA and Neu1 are negatively regulated by miR-125b expression. Cellular and clinical data indicated that expression of PUMA is most negatively regulated by the expression of miR-125b. It is also important to note that *BAK1* (another potential miR-125b target) expressions remain unchanged with respect to expression of miR-125b. The expression of PUMA and Neu-1 in BxPC3-GZR cells and the BxPC3-GZR-ΔmiR-125b cells was also determined by Western blot analysis (Fig. 6C). The data indicates that the expression of PUMA is negatively regulated by the expression of miR-125b (Fig. 6C). Similar analyses of PUMA, Neu1 and *BAK1* expression were performed for the Panc-1 and Panc-1ΔmiR-125b cells. These data suggests that the PUMA and Neu1 are functional targets of miR-125b and based on their known functions likely play a role in regulating CRMP (Fig. 6D, E).

## Discussion

Recent evidence indicates a link between chemoresistance and metastatic potential of cancer cells that possess an EMT phenotype and express stem cell markers [36]. Other studies indicate that expression pattern of specific miRNAs regulate expression of genes involved in chemoresistance and metastatic potential [10], [37], [38]. To further investigate the role of miRNAs in regulating the chemoresistant and mesenchymal phenotype (CRMP), we developed a PDAC cell line model (BxPC3-GZR) of CRMP and compared its miRNA expression profile with that of an isogenically matched parental cell line (BxPC3) that possesses an epithelial phenotype. Based on q-RT-PCR validation and potential relevance to cancer we selected a molecular signature of six miRNAs that were differentially expressed in BxPC3-GZR (four with increased expression and two with decreased expression). Finally, we compared this miRNA signature with miRNA expression and gene expression of 43 advanced PDAC clinical specimens and normal pancreas tissue from the TCGA database.

To develop a chemoresistant cell line model, BxPC3 cells were transiently exposed to increasing doses of gemcitabine over a six weeks time period. In agreement with a previous study [15], the selection of a more chemoresistant cancer cell population (BxPC3-GZR) was associated with EMT and stem cell-like properties (Fig. 1). To our knowledge this is the first study to identify a miRNA signature associated with a gemcitabine induced CRMP in PDAC. Our original eight miRNA signature validated by qPCR showed that miR-125b, miR-155, miR-100, miR-21 were up regulated and miR-1246, miR-205, miR-27b, miR455-3p were down regulated. In another study miRNA profiles were compared between a PDAC cell line that is drug resistant with a cell line that is more sensitive. In that study miR-21 was shown to be up regulated and miR-200b, miR-200c, let-7b, let-7c, let-7d, and let-7e were dramatically down regulated in gemcitabine resistant cells [39]. In agreement with this previous study we showed that miR-21 is significantly up regulated in BxPC3-GZR, our CRMP model. Further, miRNAs of the miR-200 family (miR-200a, b, c, miR-141 and miR-429) and miR-205 have been identified as key negative regulators of both EMT and the metastatic potential of cancer cells. The miR-200 family targets the key regulators of EMT including Zeb1 and Sip1 (also known as Zeb2), leading to increased E-cadherin expression levels [40], [41].

MiR-125b that is over-expressed in BxPC3-GZR model was recently reported to play a role in chemoresistance in breast cancer and as a biomarker for screening non-small-cell lung cancer as well as a diagnostic or prognostic biomarker for advanced NSCLC patients receiving cisplatin-based chemotherapy [42]–[44]. This latter finding prompted us to further assess the role of miR-125b in regulating the CRMP in BxPC3-GZR model.

We sought to establish whether this miRNA signature associated with the CRMP could differentiate distinct subpopulations of cells in clinical specimens by assessing the miRNA expression profile from 43 clinical pancreatic cancer specimens from the TGCA database. Five of the six miRNAs (four up regulated and two down regulated) showed similar levels of deregulation in the advanced PDAC tumor specimens compared to normal pancreas tissues (Fig. 2). Only miR-155 that was up regulated in the BxPC3-GZR model showed a more equal distribution of being either up or down regulated in the tumor specimens. Out of the 43-pancreatic cancer specimens, miR-100, miR-125b (isoform 1 and 2), and miR-21 was over expressed in 41, 39 and 31 tumor specimens, respectively. Of the two miRNAs down regulated in BxPC3-GZR, analyses of the clinical specimens showed that miR-27b and miR-455 were down regulated in 36 and 32 specimens, respectively (*Table 2*). The results clearly suggest a commonality in the deregulation of five of the six miRNAs found in the BxPC3-GZR model with advanced human PDAC specimens. Future studies will be needed to determine whether this five-miRNA profile is related to patient survival or response to therapy. Moreover, these five miRNAs or their target genes may have potential as novel therapeutic targets.

Because of the broad range of possible roles of miR-125b in oncogenesis and since there are limited studies of this miRNA in PDAC, we chose to further determine its functional significance in relation to CRMP. Interestingly, screening of additional PDAC cell lines showed that miR-125b correlated directly with an EMT phenotype (Fig. 3 and 4). Inhibiting the expression of miR-125b partially reversed the EMT phenotype, reduced the invasiveness and partially restored response to gemcitabine suggesting a direct role for miR-125b in maintenance of the CRMP. This result is consistent with the recent finding that miR-125b plays a role in chemoresistance of breast cancer [45], [46]. However, other studies suggest a role for miR-125b as tumor suppressor in cancers of the ovary, bladder and breast, as well as in hepatocellular carcinoma, melanoma, squamous cell carcinoma and osteosarcoma [31],[47], [48]. In contrast to the tumor-suppressive properties mentioned above, the members of miR-125 family, especially miR-125b, also act as oncogene in several cancers including prostate cancer [31], [49].

Targeting miR-125b has potential to reverse the CRMP. As mentioned above, miR-125b is deregulated in many differentiated cancer cells. For example, among 328 known and 152 novel human microRNAs, miR-125b was one of the most down-regulated microRNAs in prostate cancer [13]. Our present data demonstrated that in PDAC cells miR-125b is up regulated in both EMT and chemoresistance phenomena by attenuating expression of PUMA and Neu-1.

PUMA is known to be important in chemotherapy induced apoptosis suggesting that down regulation of PUMA promotes chemoresistance [50]. Luciferase assay including rescue experiments with mutated seed sequence of 3′UTR of PUMA was used to establish the direct involvement of miR-125b to target mRNA of PUMA and represses its expression [28], [49]. Neu1 plays an important role in regulating invasion and metastasis in different cancer types including human colon cancer and is reported to be a direct target of miR-125b [49]. A negative correlation was observed in invasion with the over-expression of Neu1 [34]. Our present data indicate that knockdown of miR-125b expression induced the up-regulation of Neu1 expression. A study in colon cancer indicates that it is a tumor suppressor gene and plays role in reducing invasive phenotype [34]. However, a separate study indicates that Neu1 facilitates EGFR signaling in pancreatic cancer and thus could have a tumor promoting effect [35]. Thus further studies are needed to weigh the effects of Neu1 effects on inhibiting invasion versus its role in EGFR signaling.

In summary we identified a set of six miRNAs that are deregulated in a PDAC model of CRMP. Analyses of 43 clinical specimens of PDAC from the TCGA data set revealed that five of the six miRNAs were commonly also deregulated in PDAC. One of the miRNAs, miR-125b, was further studied on the bases of its potential role in chemoresistance and inhibiting the expression of miR-125b partially reversed the EMT phenotype and enhanced response to chemotherapy. In addition, we found that several miR-125b target genes were down regulated and two of these *BBC3* (PUMA) and Neu1 are known tumor suppressors. We further found that knockdown of miR-125b restored the expression of PUMA and Neu1 and partially reversed CRMP. The present study identified a set of five miRNAs that are deregulated in PDAC cells showing a CRMP and in advanced PDAC tumor specimens. These miRNAs or their molecular targets may serve as the basis of new therapeutic strategies for overcoming gemcitabine resistance.

## Supporting Information

Figure S1 **Response of BxPC3 and BxPC3-GZR cells to paclitaxel were compared by MTT assays.** Cells were treated with indicated concentrations of paclitaxel and MTT assays were performed after 96 hours. A graph of the data shows only a modest reduction of sensitivity of BxPC3-GZR cells to paclitaxel as compared to control BxPC3 cells. Bars represent mean +/−SE.
(DOCX)
