# Supplementary material for: A miRNA Signature of Chemoresistant Mesenchymal Phenotype Identifies Novel Molecular Targets Associated with Advanced Pancreatic Cancer
Source: PLoS One. 2014 Sep 3;9(9):e106343. doi: 10.1371/journal.pone.0106343 (PMC4153643; doi:10.1371/journal.pone.0106343)
Supplement: Figure S1 — Response of BxPC3 and BxPC3-GZR cells to paclitaxel were compared by MTT assays. Cells were treated with indicated concentrations of paclitaxel and MTT assays were performed after 96 hours. A graph of the data shows only a modest reduction of sensitivity of BxPC3-GZR cells to paclitaxel as compared to control BxPC3 cells. Bars represent mean +/−SE. (DOCX) [file pone.0106343.s001.docx]

**Paclitaxel (dose-nM- 96h)**

**% Growth of control**

Fig S1. Response of BxPC3 and BxPC3-GZR cells to paclitaxel were compared by MTT assays. Cells were treated with indicated concentrations of paclitaxel and MTT assays were performed after 96 hours. A graph of the data shows only a modest reduction of sensitivity of BxPC3-GZR cells to paclitaxel as compared to control BxPC3 cells. Bars represent mean +/-SE.
